# Assessment of the Relationship between Oral Health Behavior, Oral Hygiene, and Gingival Status of Adolescent Tobacco Consumers in Ranchi, Jharkhand: A Comparative Study

**DOI:** 10.1155/2021/3548132

**Published:** 2021-09-09

**Authors:** Sandeep Kumar, Arunoday Kumar, Anjali Gupta, Siddharth Kumar Singh, Abhishek Gupta, Palkin Mehta

**Affiliations:** ^1^Department of Public Health Dentistry, Dental Institute, RIMS, Ranchi, Jharkhand 834009, India; ^2^Department of Prosthodontics and Crown and Bridge, Dental College, RIMS, Imphal, Manipur 795004, India; ^3^Department of Dentistry, Saraswati Medical College, Unnao, Uttar Pradesh 209859, India; ^4^Department of Oral Medicine and Radiology, Saraswati Dental College, Lucknow 227105, India; ^5^Department of Oral Medicine and Radiology, Chitwan Medical College, Bharatpur 44207, Nepal

## Abstract

**Background:**

Tobacco consumption is very prevalent in India and associated with a number of oral health problems. Good oral health behavior plays a significant role in improving oral hygiene status.

**Objectives:**

To assess the relationship between the oral health behavior, oral hygiene, and gingival status of adolescent tobacco consumers (smoke/smokeless form) and to compare it with that of the nonconsumers of tobacco in the same age group, who were selected from the OPD of Dental Institute, RIMS.

**Methods:**

This was a cross-sectional study carried out in Ranchi. The study included a total of 400 adolescents who were reported to be consumers of tobacco and 400 adolescents who were nonconsumers of tobacco. The oral health behavior was assessed using HU-DBI. The plaque and gingival scores were assessed using standardized indices. Data were analyzed using the Chi-square test, independent sample *t*-test, and Pearson's correlation. The significance level was set at *p* ≤ 0.05.

**Results:**

The majority of tobacco consumers were found to have poor plaque scores as assessed using the plaque index. As assessed by the gingival index, the majority of the tobacco consumers were found to have a severe form of gingivitis. The mean plaque score (2.38 ± 0.51, *p* value <0.001) and the mean gingival score (2.6 ± 0.63, *p* value <0.001) were significantly higher in tobacco consumers. The mean HU-DBI score was significantly higher in non-tobacco consumers (8.3 ± 1.60, *p* value <0.001). It was observed that the gingival and plaque scores have a significant negative correlation with the HU-DBI score. The majority of tobacco consumers were worried about the staining of teeth and bleeding from gums. A dental visit for a routine preventive check-up was reported to be rare in both groups.

**Conclusion:**

The oral hygiene and gingival status were significantly poor in tobacco consumers compared to non-tobacco consumers. As the oral health behavior of the participants improved, the plaque and gingival scores reduced significantly.

## 1. Introduction

Oral health plays an important role in overall health and is an indispensable part of general health [1]. The status of oral and general health depends on a dynamic interplay of several factors, including the individual's personal attributes, behaviors, and perceptions [2]. Dental health is affected by a person's oral health behaviors and oral habits, including tooth brushing, use of dental floss, and regular dental visits [3]. People's oral health knowledge, behavior, and status are influenced by many factors including culture, environment, and social customs [4, 5]. Oral health status is an essential component of oral health behavior. The term “oral health behavior” describes the complex effect on the individual oral health of the oral hygiene habits, nutritional preferences, and pattern of a person's utilization of dental services [6, 7].

Tobacco consumption is very prevalent in India and associated with a number of oral premalignant and malignant lesions as reported by a number of studies [8–10]. The consumption of tobacco either in smoke or smokeless form is a major public health challenge and is initiated by many from an early age. Among the Indian studies, the mean age of initiation of tobacco use has been found to vary from 8 to 15 years [11–13]. The duration of tobacco consumption is an important predictor for the development of oral malignant lesions, thereby putting individuals initiating the habit at the early stages of life (adolescents) at a higher risk [8, 14, 15]. Adolescents often tend to neglect their oral health due to lack of training and poor understanding of the importance of oral health for general health. Hence, there is a need to create awareness pertaining to oral health among adolescents and provide them with adequate training. The motivation to improve oral hygiene needs to be implemented from the early stages.

Limited studies have been carried out assessing the relationship between oral health behavior, oral hygiene, and gingival status in tobacco consumers. Since the development of healthy oral health behavior and improvement in oral hygiene status need to be inculcated from the early stages of life (adolescents), this study was carried out with an aim to assess the relationship between the oral health behavior, oral hygiene, and gingival status of adolescent tobacco consumers (smoke/smokeless form) and to compare it with that of the nonconsumers of tobacco in the same age group who were selected from the OPD of Dental Institute, RIMS. The study findings will enable policymakers to draft effective policies for the improvement of oral health status in adolescents and also plan effective training programs to create awareness pertaining to oral diseases.

## 2. Material and Methods

This was a cross-sectional study carried out by the Department of Public Health Dentistry, Dental Institute, RIMS, Ranchi. The study included a total of 400 adolescents who were consumers of tobacco and who had visited Dental Institute, RIMS, for some form of treatment. The relatives/friends of patients who had the habit of tobacco consumption and had accompanied the patients to OPD were also included in the study. A similar pattern was employed to select 400 adolescents who were nonconsumers of tobacco that served as a comparison group. Hence, 800 participants took part in the study. The participants were selected using a convenient sampling technique. The Institutional Ethics Committee, Rajendra Institute of Medical Sciences, Ranchi, granted ethical clearance for conducting the study. Participation in the study was voluntary. Verbal informed consent was sought from the participants. The response rate was 99.0%.

The individuals who consented to participate in the study were in adolescent age, gave a history of tobacco consumption, were not using any orthodontic appliances, and did not suffer from any systemic diseases which contradict oral examinations included in the study. The rest were excluded from the study. A similar pattern was employed to select a comparison group with the only difference that the individuals in the comparison group were nonconsumers of tobacco.

The sample size calculation was based upon the findings of a pilot study that was carried out on 25 individuals who were consumers and nonconsumers of tobacco. Keeping the power of the study at 80%, alpha error = 0.05, and applying the formula for sample size calculation as recommended by WHO, [16] we found that a minimum sample of 384 in each group will be required. It was rounded off to 400 participants in each group. Hence, the final sample size for the study was 800 individuals.

A questionnaire that collected information on sociodemographic characteristics was drafted. It also comprised 22 questions that assessed attitude and behavior towards oral health. The oral hygiene status was assessed using the plaque index whereas the gingival status was assessed using the gingival index. The oral health behavior was assessed using the Hiroshima University-Dental Behavioral Inventory (HU-DBI). This inventory was developed by Kawamura et al. [17]. It has shown good test-retest reliability and well-translated validity as reported by a number of studies [7, 18]. It was translated into the local Hindi language. The validity of the questionnaire was checked using a back-translation method involving blind retranslations into English. The validity of translation was verified by experts in both languages. The responses of the questions of this inventory were dichotomized into “agree” and “disagree.” For questions number 4, 9, 11, 12, 16, and 19, one point is given for each “agree” response to items. For questions number 2, 6, 8, 10, 14, and 15, one point is given for each “disagree” response to items. Higher scores in HU-DBI indicated better oral health.

The assessment of socioeconomic status was done using a modified Kuppuswamy scale [19]. After data collection, it was further categorized into three strata of the upper, middle, and lower class for the sake of data analysis.

The participants who fulfilled the inclusion criteria were asked to fill a pro forma. The filled pro forma were collected and checked for any incomplete responses. Any doubts arising during the filling of the pro forma was clarified by the investigator himself. The entire data collection for the study was carried out under the supervision of one trained examiner. The assessment of the plaque score was done as per the guidelines of the plaque index (1964) given by Loe [20]. Six index teeth were examined using standard protocols. If any of the index teeth was missing, then the entire dentition was evaluated. The armamentarium used was a mouth mirror, light source, and explorer. The assessment of the gingival score was done as per the guidelines of the gingival index (1963) given by Loe [20]. Six index teeth were examined using standard protocols. If any of the index teeth was missing, then the entire dentition was evaluated. The armamentarium used was a mouth mirror, light source, and Williams' periodontal probe.

All statistical tests were performed using SPSS v 20. The data were analyzed for frequency distribution. Chi-square test, independent sample *t*-test, and Pearson's correlation analysis were performed. *p* value <0.05 was considered statistically significant.

## 3. Results

No significant differences (*p* value >0.05) were observed with respect to age, gender, socioeconomic status, and place of residence between tobacco and non-tobacco consumers **(**Table 1**)**.

The majority of the tobacco consumers were reported to have a fair (30.00%) or poor (45.00%) plaque scores whereas the majority of the non-tobacco consumers had an excellent (37.50%) or good (35.00%) plaque scores, and there was a significant difference (*p* value <0.001) observed in the plaque score between the two groups. The majority of the tobacco consumers reported suffering from severe (50.00%) to moderate (32.50%) gingivitis whereas the majority of non-tobacco consumers had mild (40.00%) to moderate (40.00%) gingivitis, and there was a significant difference (*p* value <0.001) observed in the gingival score between the two groups (Table 2).

A significant difference (*p* value >0.05) was observed with the majority (69.50%) of tobacco consumers reporting bleeding gums while brushing. Moreover, they reported finding white sticky deposits (70.75%) on teeth and were worried about the color of their teeth (55.00%). A significantly higher proportion of tobacco consumers were bothered about the color of their gums (78.25%), believed that their teeth are getting worse despite daily brushing (79.75%), and were of the opinion that they cannot help having false teeth when they grow old (83.00%). A significantly higher proportion of tobacco consumers complained of halitosis (69.25%) **(**Table 3).

The tobacco consumers reported having a significantly higher (*p* value <0.001) mean plaque and gingival scores compared to non-tobacco consumers. The non-tobacco consumers reported having a significantly higher (*p* value <0.001) mean HU-DBI score compared to tobacco consumers (Table 4).

A significant negative correlation was observed between the plaque score and HU-DBI score (*r* = −0.503, *p* value <0.001) and between the gingival score and HU-DBI score (*r* = −0.559, *p* value <0.001) in the study population. A significant positive correlation (*r* = 0.389, *p* value <0.001) was observed between the plaque score and gingival score of the study population (Table 5**).**

## 4. Discussion

The present study was carried out with the objective of assessing the relationship between oral health behavior, oral hygiene, and gingival status of adolescent tobacco consumers in Ranchi, Jharkhand. The secondary objective was to compare the oral health behavior, oral hygiene, and gingival status of tobacco consumers and nonconsumers. No significant differences were observed with respect to age, gender, socioeconomic status, and place of residence between tobacco and non-tobacco consumers. This indicates that the population under study was homogenous, and this enables us to minimize bias due to uneven distribution of the study population.

Tobacco is known to have ill effects on oral health [21, 22]. Tobacco has many negative effects on the mouth, including staining of teeth and dental restorations, reduction of the ability to smell and taste, and development of oral diseases such as smoker's palate; smoker's melanosis; coated tongue; and, possibly, oral candidiasis and dental caries, periodontal disease, implant failure, oral precancer, and cancer [23]. In the present study, a number of participants in the tobacco consumer group demonstrated a severe form of gingivitis and had a higher mean gingival score compared to non-tobacco consumers. This is similar to the findings reported by Petrovic et al. wherein they found that the smokers group had higher gingival bleeding. It was also found that the majority of the participants in the tobacco consumer group had poor plaque and had higher mean plaque scores compared to non-tobacco consumers [24]. This is indicative of the fact that an increased amount of dental plaque in tobacco consumers is because of lower oral hygiene. Wilson in his study had stated that an increased amount of dental plaque was a consequence of smoking dental deposits [25].

The assessment of oral health behavior was done using HU-DBI. A higher score on the inventory indicates better oral health behavior. The mean HU-DBI score was found to be significantly higher in non-tobacco consumers compared to tobacco consumers. This is suggestive of the fact that non-tobacco consumers had better oral hygiene awareness and a positive attitude towards oral health. The consumption of tobacco has a deleterious effect on oral health and impairs oral health-related quality of life [26]. The tobacco consumers were found to lack good oral health behavior and demonstrated a negative attitude towards oral hygiene awareness.

As per the findings of the HU-DBI, a significantly higher number of tobacco consumers reported noticing white deposits on their teeth and were worried about the color of their teeth. The consumption of tobacco leads to staining of teeth as reported by various studies [22, 27], and a stained surface provides a medium for the accumulation of plaque and white deposits. Studies have also reported that calculus formation is faster in tobacco consumers, due to increased salivary secretions and more amount of calcium in the secreted saliva immediately after tobacco consumption [28].

A significantly higher number of tobacco consumers reported bleeding of gums. In a study done by Mavropoulos et al., it was concluded that small recurrent vasoconstrictive attacks, during a long period of smoking and tobacco consumption, could lead to gingival vascular dysfunction and periodontal disease. Induced vasoconstriction could impair gingival blood vessels and could reduce the amount of oxygen and blood elements that supply gingiva with nutritive elements [29, 30].

A significantly higher number of tobacco consumers were bothered about the color of their gums, were of the opinion that their teeth are getting worse despite daily tooth brushing, and thought that they cannot help having false teeth when they grow old. Tobacco consumption has deleterious effects on oral health leading to a number of oral premalignant and malignant lesions, periodontal crippling, and ultimately loss of teeth [8, 31].

Although without statistical significance, very few reported visiting dentists for regular check-ups, and they were never taught professionally to brush their teeth, did not use floss, or were aware of oral hygiene maintenance. In India, dental visit for preventive care is very rare, and people often visit dentists only when there is an emergency dental need. This is termed the “healthy person non-visitors effect” and has been reported in a number of Indian studies [8, 32]. In India, the use of floss, mouth rinses, and other advanced methods for plaque removal is not in practice by the general population, and traditional methods like twigs, datun, and others are still the preferred choice for oral hygiene maintenance. This can be attributed to the fact that these traditional items for oral care are cheap and readily available. These findings also emphasize the need to improve oral hygiene awareness among the population.

In the present study, it was observed that the plaque and gingival scores have a significant negative correlation with the HU-DBI score. Similar findings were reported in a study carried out by Lalani et al. [7] and Rahman and Al Kawas [6]. This is indicative of the fact that participants who had higher HU-DBI scores had lower plaque and gingival scores. As the oral health behavior improved and participants demonstrated a positive attitude towards oral health maintenance, the plaque and gingival score significantly reduced. Thus, this study suggests an association between oral health behavior, oral hygiene, and gingival status.

Some of the limitations of the study include the study design. It was cross-sectional in nature which allowed the identification of trends but cannot explain the causation of changes over time in attitudes and behavior of participants. There are chances for social desirability bias as some of the participants may not have been comfortably provided with the correct frequency and duration of tobacco consumption. In addition to these, there are certainly other factors like psychological and emotional factors that were not evaluated in this study. Further, longitudinal studies on a larger sample size need to be carried out before the results can be generalized.

## 5. Conclusion

The oral hygiene and gingival status were significantly poor in tobacco consumers compared to non-tobacco consumers. The mean HU-DBI score was significantly higher in non-tobacco consumers. It was observed that the plaque score and gingival score have a significant negative correlation with the HU-DBI score. A significant positive correlation was observed between the plaque score and gingival score of the study population. The majority of the tobacco consumers reported bleeding gums, observed white sticky deposits on their teeth, and reported staining of their teeth. They were also bothered about the color of their gums, believed their teeth are getting worse despite daily brushing, and were of the opinion that they cannot help having false teeth when they grow old. A significantly higher proportion of tobacco consumers also complained of halitosis. In both groups, dental visit for a preventive check-up was reported to be very rare.

## Figures and Tables

**Table 1:** Sociodemographic characteristics of study population.

Variables	Categories	Tobacco consumers	Non-tobacco consumers	*p* value
Mean age		16.59 ± 1.75	16.60 ± 1.70	0.951

Gender	Male	225 (56.25%)	240 (60.00%)	0.315
	Female	175 (43.75%)	160 (40.00%)	

Socioeconomic status	Lower class	170 (42.5%)	154 (38.5%)	0.514
	Middle class	150 (37.5%)	161 (40.25%)	
	Upper class	80 (20.00%)	85 (21.25%)	

Residence	Urban	185 (46.25%)	167 (41.75%)	0.199
	Rural	215 (53.75%)	233 (58.25%)	

*p* value ≤0.05: statistically significant difference.

**Table 2:** Distribution of plaque and gingival scores in tobacco and non-tobacco consumers.

Factors	Categories	Tobacco consumers	Non-tobacco consumers	*p* value (chi-square test)
Plaque score	Excellent	70 (17.50%)	150 (37.50%)	<0.001^*∗*^
	Good	30 (7.50%)	140 (35.00%)	
	Fair	120 (30.00%)	50 (12.50%)	
	Poor	180 (45.00%)	60 (15.00%)	

Gingival score	Mild	70 (17.50%)	160 (40.00%)	<0.001^*∗*^
	Moderate	130 (32.50%)	160 (40.00%)	
	Severe	200 (50.00%)	80 (20.00%)	

^*∗*^*p* value ≤0.05: statistically significant difference.

**Table 3:** HU-DBI questionnaire items and percentage of “agree”/“disagree” responses.

Items	Tobacco consumers		Non-tobacco consumers		*p* value
	Agree	Disagree	Agree	Disagree	
I do not worry much about visiting the dentist	324 (81.00%)	76 (19.00%)	328 (82.00%)	72 (18.00%)	0.715
My gums tend to bleed when I brush my teeth	278 (69.50%)	122 (30.50%)	123 (30.75%)	277 (69.25%)	<0.001^*∗*^
I worry about the color of my teeth	220 (55.00%)	180 (45.00%)	85 (21.25%)	315 (78.75%)	<0.001^*∗*^
I have noticed some white sticky deposits on my teeth	283 (70.75%)	117 (29.25%)	80 (20.00%)	320 (80.00%)	<0.001^*∗*^
I use a child size toothbrush	213 (53.25%)	187 (46.75%)	216 (54.00%)	184 (46.00%)	0.831
I think that I cannot help having false teeth when I am old	332 (83.00%)	68 (17.00%)	153 (38.25%)	247 (61.75%)	<0.001^*∗*^
I am bothered by the color of my gums	313 (78.25%)	87 (21.75%)	97 (24.25%)	303 (75.75%)	<0.001^*∗*^
I think my teeth are getting worse despite my daily brushing	319 (79.75%)	81 (20.25%)	123 (30.75%)	277 (69.25%)	<0.001^*∗*^
I brush each of my teeth carefully	327 (81.75%)	73 (18.25%)	311 (77.75%)	89 (22.25%)	0.159
I have never been taught professionally how to brush	383 (95.75%)	17 (04.25%)	377 (94.25%)	23 (05.75%)	0.330
I think I can clean my teeth well without using a toothpaste	133 (33.25%)	267 (66.75%)	132 (33.00%)	268 (67.00%)	0.940
I often check my teeth in a mirror after brushing	383 (95.75%)	17 (4.25%)	374 (93.50%)	26 (6.50%)	0.158
I worry about having a bad breath	277 (69.25%)	123 (30.75%)	83 (20.75%)	317 (79.25%)	<0.001^*∗*^
It is impossible to prevent gum disease with tooth brushing alone	213 (53.25%)	187 (46.75%)	215 (53.75%)	185 (46.25%)	0.887
I put off going to the dentist until I have toothache	64 (16.00%)	336 (84.00%)	81 (20.25%)	319 (79.75%)	0.118
I have used a dye to see how clean my tooth are	0 (0.00%)	400 (100.00%)	0 (0.00%)	400 (100.0%)	1.000
I use a toothbrush with hard bristles	20 (5.00%)	380 (95.00%)	21 (5.25%)	379 (94.75%)	0.872
I do not feel I have brushed well unless I brush with strong strokes	143 (35.75%)	257 (64.25%)	134 (33.50%)	266 (66.50%)	0.508
I feel I sometimes take too much time to brush my teeth	113 (28.25%)	287 (71.75%)	119 (29.75%)	281 (70.25%)	0.640
I have had my dentist tell me that I brush very well	0 (0.00%)	400 (100.00%)	0 (0.00%)	400 (100.00%)	1.000
I do use tooth floss on regular basis	20 (5.00%)	380 (95.00%)	21 (5.25%)	379 (94.75%)	0.872
I brush my teeth twice daily or more	151 (37.75%)	249 (62.25%)	153 (38.25%)	247 (61.75%)	0.884

^*∗*^*p* value ≤0.05: statistically significant difference.

**Table 4:** Comparison of mean plaque score, gingival score, and HU-DBI score between tobacco and non-tobacco consumers.

Factors	Tobacco consumers	Non-tobacco consumers	*p* value
Mean plaque scores	2.38 ± 0.51	0.93 ± 0.22	<0.001^*∗*^
Mean gingival scores	2.6 ± 0.63	0.91 ± 0.18	<0.001^*∗*^
Mean HU-DBI scores	3.8 ± 0.89	8.3 ± 1.60	<0.001^*∗*^

^*∗*^*p* value ≤0.05: statistical significant difference.

**Table 5:** Correlation between plaque score, gingival score, and HU-DBI score.

Variables	Correlation coefficients	*p* value
Plaque scores-HU-DBI scores	−0.503	<0.001^*∗*^
Gingival score-HU-DBI score	−0.559	<0.001^*∗*^
Plaque score-gingival score	0.389	<0.001^*∗*^

^*∗*^*p* value ≤0.05: statistically significant difference.

## Data Availability

The data used to support the findings of this study may be available from the corresponding author upon request.

## References

[1] Baiju R. M., Peter E., Varghese N. O., Sivaram R. (2017). Oral health and quality of life: current concepts. *Journal of Clinical Diagnostic Research*.

[2] Atchison A. K., Dubin L. F. (2003). Understanding health behavior and perceptions. *Dental Clinics North America*.

[3] Hsu K. J., Yen Y. Y., Lan S. J., Wu Y. M., Lee H. E. (2013). Impact of oral health behaviors and oral habits on the number of remaining teeth in older Taiwanese dentate adults. *Oral Health Preventive Dentistry*.

[4] Ivica A., Galic N. (2014). Attitude towards oral health at various colleges of the university of zagreb: a pilot study. *Actastomatologica Croatica*.

[5] Nyamuryekung’e K. K., Lahti S. M., Tuominen R. J. (2018). Attitudes towards tooth fillings in Tanzanian adults and its association with previous filling experience. *BMC Oral Health*.

[6] Rahman B., Al Kawas S. (2013). The relationship between dental health behavior, oral hygiene and gingival status of dental students in the United Arab Emirates. *European Journal of Dentistry*.

[7] Lalani A., Dasar P. L., Sandesh N., Mishra P., Kumar S., Balsaraf S. (2015). Assessment of relationship between oral health behavior, oral hygiene and gingival status of dental students. *Indian Journal of Dental Research*.

[8] Kumar S., Debnath N., Ismail M. B. (2015). Prevalence and risk factors for oral potentially malignant disorders in Indian population. *Advance in Preventive Medicine*.

[9] Pahwa V., Nair S., Shetty R. S., Kamath A. (2018). Prevalence of oral premalignant lesions and its risk factors among the adult population in Udupi taluk of coastal Karnataka, India. *Asian Pacific Journal of Cancer Prevention*.

[10] Balsaraf S., Bhambal A., Chole R. (2019). Study of oral potentially malignant disorders related to various risk factors amongst the patients attending hospitals in Bhopal. *India Medicine Pharmaceutical Representative*.

[11] Bhojani U. M., Chander S. J., Devadasan N. (2009). Tobacco use and related factors among pre-university students in a college in Bangalore, India. *National Medical Journal of India*.

[12] Chadda R. K., Sengupta S. N. (2002). Tobacco use by Indian adolescents. *Tobacco Induced Diseases*.

[13] Kapil U., Goindi G., Singh V., Kaur S., Singh P. (2005). Consumption of tobacco, alcohol and betel leaf amongst school children in Delhi. *Indian Journal of Pediatrics*.

[14] Aishwarya K. M., Reddy M. P., Kulkarni S., Doshi D., Reddy B. S., Satyanarayana D. (2017). Effect of frequency and duration of tobacco use on oral mucosal lesions—a cross-sectional study among tobacco users in Hyderabad, India. *Asian Pacific Journal of Cancer Prevention*.

[15] Garg K. N., Raj V., Chandra S. (2013). Trends in frequency and duration of tobacco habit in relation to potentially malignant lesion: a 3 year’s retrospective study. *Journal of Oral Maxillofac Pathol*.

[16] World Health Organization (1999). *Oral Health Surveys, Basic Methods*.

[17] Kawamura M., Ikeda‐Nakaoka Y., Sasahara H. (2020). An assessment of oral self‐care level among Japanese dental hygiene students and general nursing students using the hiroshima University—dental behavioural inventory (HU‐DBI): surveys in 1990/1999. *European Journal of Dental Education*.

[18] Hashim R., Ibrahim M. (2013). Oral health attitudes and behavior among dental students in Ajman, United Arab Emirates. *Journal of International Dental Medical Research*.

[19] Kumar N., Shekhar C., Kumar P., Kundu A. S. (2007). Kuppuswamy’s socioeconomic status scale-updating for 2007. *Indian Journal of Pediatrics*.

[20] Loe H. (1967). The gingival index, the plaque index and the retention index systems. *Journal of Periodontol*.

[21] Komar K., Glavina A., Boras V. V., Verzak Z., Brailo V. (2018). Impact of smoking on oral health: knowledge and attitudes of croatian dentists and dental students. *Acta Stomatologica Croatica*.

[22] Mubeen K., Chandrashekhar H., Kavitha M., Nagarathna S. (2013). Effect of tobacco on oral-health an overview. *Journal of Evolution of Medical and Dental Sciences*.

[23] Tobacco J. R. (2003). Oral diseases update on the evidence, with recommendations. Karger. *Medical Principles Practice*.

[24] Petrovic M., Kesic L., Savic Z. (2013). Comparative analysis of smoking influence on periodontal tissue in subjects with periodontal disease. *Mater Sociomed*.

[25] Wilson T. (1998). Effects of smoking on periodontium. *Quintessence International*.

[26] Sagtani R. A., Thapa S., Sagtani A. (2020). Smoking, general and oral health related quality of life—a comparative study from Nepal. *Health Qual Life Outcomes*.

[27] Alkhatib M. N., Holt R. D., Bedi R. (2005). Smoking and tooth discolouration: findings from a national cross-sectional study. *BMC Public Health*.

[28] Khan G. J., Salah-ud-Din M. R., Marwat F. M., Haq I., Reihman J. (2005). Secretion of calcium in the saliva of long term tobacco users. *Journal of Ayub Medical College Abbottabad*.

[29] Mavropoulos A., Aars H., Brodin P. (2003). Hyperaemic response to cigarette smoking in healthy gingiva. *Journal of Clinical Periodontology*.

[30] Bergstrom J., Persson L., Preber H. (1988). Influence of cigarette smoking on vascular reaction during experimental gingivitis. *Scandinavian Journal of Dental Research*.

[31] Patil P. B., Bathi R., Chaudhari S. (2013). Prevalence of oral mucosal lesions in dental patients with tobacco smoking, chewing, and mixed habits: a cross-sectional study in South India. *Journal of Family Community Medicine*.

[32] Okunseri C., Chattopadhyay A., Lugo R. I., McGrath C. (2005). Pilot survey of oral health-related quality of life: a crosssectional study of adults in Benin city, Edo state, Nigeria. *BMC Oral Health*.

